# Antibacterial and antibiofilm activity of novel nanofibers bandage formulated with *Juniperus communis* essential oil targeting antibiotic resistant bacterial strains

**DOI:** 10.1016/j.chmed.2026.05.005

**Published:** 2026-05-08

**Authors:** Mohannad N. AbuHaweeleh, Ahmad Hamdan, Menatalla Metwally Said, Maryam Hassiba, Robin Augustine, Anwarul Hassan, Nahla O. Eltai, Susu M. Zughaier

**Affiliations:** a Department of Basic Medical Sciences, College of Medicine, QU Health, Qatar University, Doha 2713, Qatar; b Department of Radiology, Stanford Medicine, Stanford University, CA 94304, USA; c Department of Mechanical and Industrial Engineering, College of Engineering, Qatar University, Doha 2713, Qatar; d Biomedical Research Center, QU Health, Qatar University, Doha 2713, Qatar

**Keywords:** antimicrobial resistance, ESKAPE pathogens, essential oil, *Juniper communis* L., nanofiber bandages

## Abstract

**Objective:**

The alarming increase in antimicrobial resistance (AMR) threatens current armament of antibiotics. This global threat to public health revived the search for plant-derived antimicrobial therapeutics. *Juniperus communis* L. has been used in folk medicine for centuries. This study aims to examine the antibacterial and antibiofilm activity of polycaprolactone (PCL) nanofiber bandages formulate with essential oil extracted from *J. communis* against clinically relevant ESKAPE (*Escherichia coli*, *Enterococcus faecium*, *Staphylococcus aureus*, *Klebsiella pneumoniae*, *Acinetobacter baumanii*, *Pseudomonas aeruginosa*, and *Enterobacter cloacae*) bacterial pathogens commonly causing nosocomial infections.

**Methods:**

Bacterial growth curves and bactericidal assays were performed on antibiotic sensitive and resistant clinical bacterial strains collectively known as ESKAPE pathogens. Antibiofilm activity of Juniper essential oil (JEO) was tested against methicillin resistant *S. aureus* (MRSA) and *E. coli* individually in a 96-well plate, and the 96 well plate which contains the bacteria was incubated over night to allow biofilm formation and stained using crystal violet. The antibacterial activity of PCL-based nanofiber membrane formulated with various doses (2%, 4%, 6%, and 8%) of JEO was tested using disc diffusion method.

**Results:**

JEO dilutions (50, 25 and 12.5 µL) demonstrated a dose-dependent antibacterial activity against tested strains and significantly reduced growth after 24 h. JEO also exerted antibiofilm activity against MRSA and *E. coli* strains that are resistant to antibiotics. Additionally, PCL nanofiber membranes formulated with 8% JEO inhibited MRSA growth as demonstrated by large zone of inhibition on agar plate.

**Conclusion:**

JEO has antibacterial activity against ESKAPE pathogens. The potential application of JEO formulated in PCL nanofiber bandages is for wound dressing to treat surgical site infections, burns or skin ulcers.

## 1 Introduction

Infectious diseases are major cause of both morbidity and mortality, especially in low- or middle-income countries (Salam et al., 2023, Uchil et al., 2014). Various antimicrobial drugs have been used to treat infections, however increase in resistance to these medications is alarming. Antimicrobial resistance (AMR) has been declared, by the World Health Organization (WHO), as one of the top 10 global public health threats that could affect humanity (EclinicalMedicine, 2021). AMR is a major global concern since it has high death rates, increased health care costs, and decreased health care productivity (Uchil et al., 2014). AMR is mostly due to misuse and overuse of antimicrobials, which lead to the development of drug-resistant pathogens (EClinicalMedicine, 2021, Salam et al., 2023). This crisis is highly censorious in nosocomial infections caused by the ESKAPE pathogens: *Escherichia coli*, *Enterococcus faecium*, *Staphylococcus aureus*, *Klebsiella pneumoniae*, *Acinetobacter baumanii*, *Pseudomonas aeruginosa*, and *Enterobacter cloacae* (Santajit & Indrawattana, 2016). Antimicrobial therapies are becoming largely ineffective as the spread of drug-resistance pathogens keeps on escalating (CDC, 2024).

This global threat to public health revived the search for plant-derived antimicrobial therapeutics (Chandra et al., 2017, Lagha et al., 2019, Palaniappan and Holley, 2010, Savoia, 2012). Over the centuries, plant-derived essential oils showed that it poses a wide spectrum of antimicrobial and antibiofilm effects on various pathogens with alleviation of the diseases’ symptoms and reduction in bacterial growth (Frydrysiak et al., 2021, Lagha et al., 2019). Essential oils have shown success in treating several conditions including analgesia associated with chronic diseases or medical operations, pediculosis in children, and postoperative nausea in cancer patients (Man et al., 2019). Biofilm formation seen in different medical devices such as catheters, ventilators, and contact lenses is a critical problem in clinical settings as it increases resistance to antimicrobials (Assefa & Amare, 2022). Hence, the use of natural therapeutic agents to eradicate these pathogens and inhibit the biofilm formation became necessary (Gómez-Sequeda et al., 2020).

Several studies have proposed multiple antibacterial and antibiofilm mechanisms for plant-derived essential oil components such as *α*-pinene, sabinene, and limonene (Melkina et al., 2021). These bioactive compounds are believed to exert their effects primarily by disrupting bacterial cell membranes, leading to increased permeability and cell lysis (Touati et al., 2025). Additionally, they interfere with quorum sensing pathways, thereby inhibiting bacterial communication essential for biofilm formation and virulence regulation (Maggio et al., 2025, Huang et al., 2024). These compounds have also been shown to destabilize the extracellular polymeric substance (EPS) matrix of biofilms, weakening biofilm structure and enhancing antibiotic penetration (Touati et al., 2025). Furthermore, suppression of key virulence gene expression aids in reducing pathogenicity and resistance. Collectively, these multifaceted mechanisms contribute to the promising antimicrobial and antibiofilm potential of terpenoid-rich essential oils in both clinical and environmental settings.

*Juniper communis* L. plant is one of the well-known species that is found extensively in temperate regions such as Europe, Asia, and North America (Pepeljnjak et al., 2005). Moreover, it has distinctive traits including diuretic, gastrointestinal irritation, anti-inflammatory, antihyperglycemic, hepatoprotective, antiseptic, antioxidant, antifertility, and hypoglycemic properties (Bais et al., 2014, Maurya et al., 2018, Raina et al., 2019). Different part of the plants demonstrates various functions against diseases (Bais et al., 2014, Raina et al., 2019). According to Banerjee et al. the methanolic extract from *J. communis* showed a dose-dependent analgesic effect on Swiss albino mice (Banerjee et al., 2012). Other studies have shown that Juniper essential oil (JEO) can be used as a water disinfectant from mycobacteria species in various water systems including pools, baths, and fresh water (Peruč et al., 2018).

There is a potential application of JEO to be formulated in nonfabric polymers that can be used to treat surgical site infections (SSIs) or skin ulcers. The aim of this study is to examine antibacterial, antibiofilm and immune modulatory activity of polycaprolactone (PCL) nanofiber bandages formulated with essential oil extracted from *J. communis* against clinically relevant ESKAPE bacterial pathogens commonly causing wound and nosocomial infections.

## 2 Materials and methods

### 2.1 Materials

Tryptic soy broth (TSB) (catalog number SKU 292770; 18 mL tubes; Becton, Dickinson and Company, Franklin Lakes, NJ, USA) was used for bacterial culture. The medium was supplied ready-to-use and quality controlled by the manufacturer. Phosphate-buffered saline (PBS) was obtained from Sigma-Aldrich (catalog number P4474; pH 7.4; sterile-filtered; suitable for cell culture; St. Louis, MO, USA). Crystal violet solution was obtained from Sigma-Aldrich (catalog number SKU HT901-8FOZ; St. Louis, MO, USA). Polycaprolactone (PCL; average molecular weight 80 000; analytical grade) was purchased from Sigma-Aldrich (St. Louis, MO, USA). 30% Acetic acid (catalog number SKU 1591660500; 500 mL; Merck KGaA, Darmstadt, Germany) was used in this study. Dimethyl sulfoxide (DMSO, 100%) was used as a solvent to dissolve JEO. DMSO was purchased from Sigma-Aldrich (Catalog No. 472301, St. Louis, MO, USA).

### 2.2 Juniper essential oil (JEO) extraction

JEO (purity > 96%) was extracted at the pharmaceutical laboratory at the University of Sassari and was a kind gift from Dr. Mathew Donadu from the Department of Biomedical Sciences, University of Sassari, Sassari, Italy. The JEO was extracted from *J. communis* berries. Details of JEO chemical composition are described previously (Angioni et al., 2003).

### 2.3 Bacterial growth curve

The antibacterial activity of JEO was tested against various strains of the ESKAPE pathogens. Clinically relevant isolates of ESKAPE pathogens were obtained from American Type Culture Collection (ATCC) (Manassas, VA). Bacterial strains were prepared by culturing them overnight on nutrient agar plates, then collecting the colonies and suspending them in TSB. Since all antibacterial testing were conducted using 96 well plates with a maximum volume of 250 µL, at starting diluting JEO of 50 µL was chose for serial fold dilutions. The dilutions (ranging from 6.25 µL to 50 µL) of the JEO were prepared in microtiter plate using PBS as a diluent and by performing two-fold dilution with a final volume of 50 µL of the oil per well. Bacterial inoculum from freshly grown cultures overnight in TSB was adjusted to absorbance (*A*) of 0.1 at 600 nm wavelength (*A*_600_ = 0.1). The adjusted bacterial inoculum (200 µL) was added to the oil dilutions in 96-well plate. These plates were incubated at 37 °C overnight. Bacterial growth kinetics were measured at the starting time of incubation, after 1, 2, 3, 4 and 24 h of incubation by reading the *A* at a wavelength of 600 nm.

### 2.4 Antibiofilm activity assay

To assess the antibiofilm activity of JEO, bacterial strains were grown overnight in 96 well plates in presence and absence of oil dilutions as described above. Green fluorescent protein (GFP)-labeled *E. coli* strain was kind gift from Dr. Layla Kamareddine, Qatar University. The microtiter plates containing the bacterial strains and different oil dilutions (ranging from 6.25 µL to 50 µL) were washed twice with 300 µL of distilled water. Each well in these plates was stained with 125 µL of 0.1% crystal violet for 25 min with gentle shaking. The plates were then rinsed three times, blotted, and left to dry upside down overnight. The next day, 30% acetic acid (125 µL) was added to each well and incubated for around 12 min. From each well, 100 µL of the dissolved suspension were transferred to a new plate for optical density (OD) measurement at 550 nm wavelength. 30% Acetic acid was used as a blank, which was subtracted from the OD readings.

### 2.5 PCL nanofabrication

PCL wound dressing was fabricated using a previously described methodology (Augustine et al., 2023). Briefly, air-jet spinning was used to fabricate PCL impregnated with different concentrations (1%, 2%, 4%, and 8%) of the JEO. These concentrations were selected for skin testing, with the aim of achieving oil dose sparing while remaining suitable for pharmaceutical formulations (Choe et al., 2023). PCL membranes were cut into pieces with equal size of 1 cm × 1 cm. To test antibacterial activity of PCL nanofibers with or without JEO, methicillin resistant *S. aureus* (MRSA) strain inoculum was adjusted to 0.5 McFarland standard (Leber, 2016) and spread evenly on sheep blood agar. PCL nanofiber pieces where then placed over the inoculated agar plate. Due to the volatile nature of the oil, a respective amount of the oil was added to each PCL membrane (e.g., 20 µL of JEO was added to the membrane coated with 2% of JEO). The tested percentages of JEO were 1%, 2%, 4%, and 8%. PCL membrane without JEO was used as a control. Zone of inhibition of bacterial growth around the membranes were observed after overnight incubation at 37 °C.

### 2.6 Statistical analysis

Statistical analysis was performed using GraphPad Prism software. Results were expressed as means ± standard deviations (SD). Comparison of different concentrations were done by ordinary one-way ANOVA. A *P* value of less than 0.05 was considered statistically significant.

## 3 Results

### 3.1 Antibacterial activity of JEO

**Fig. 1:**
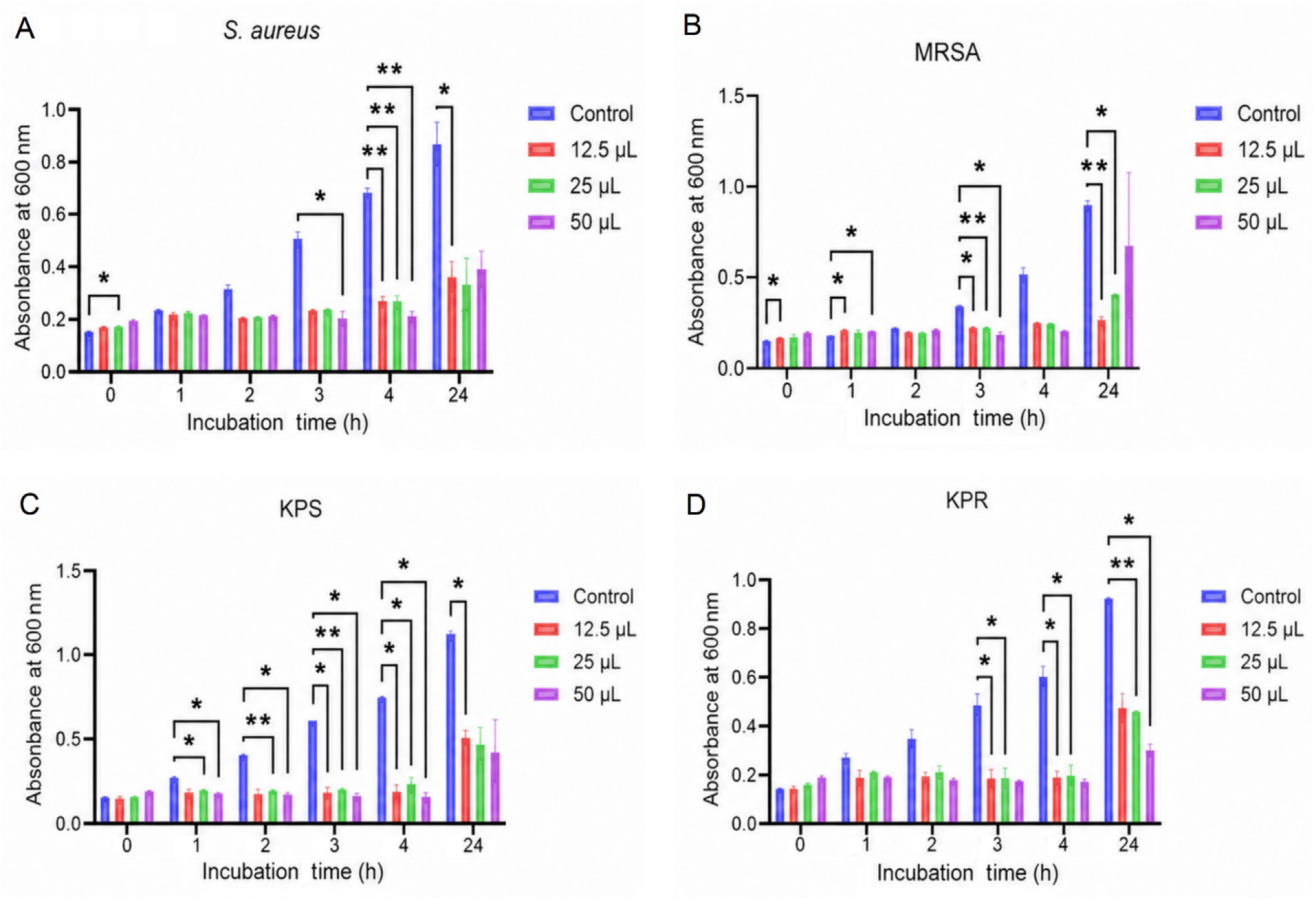
JEO inhibited the growth of Gram-positive and Gram-negative bacteria. Time course bacterial growth curves were performed. JEO inhibited bacterial growth of (A) *S. aureus,* (B) MRSA, (C) KPS, and (D) KPR*.* Each run represents the average absorbance of a duplicate readings, and the error bars represent the standard deviation. JEO: Juniper essential oil; MRSA: methicillin resistant *S. Aureus*; KPS: *K. pneumoniae* sensitive; KPR: *K. pneumoniae* resistant. Statistical analysis was performed using one-way ANOVA, *P < 0.05, ^**^P < 0.01 *vs* control group.

The antibacterial activity of JEO doses (50, 25 and 12.5 µL) was demonstrated and resulted in growth inhibition of Gram-positive *S. aureus*, MRSA, and Gram-negative *K. pneumoniae* sensitive (KPS) and resistant (KPR) strains over a duration of 1, 2, 3, 4 and 24 h (Fig. 1).

**Fig. 2:**
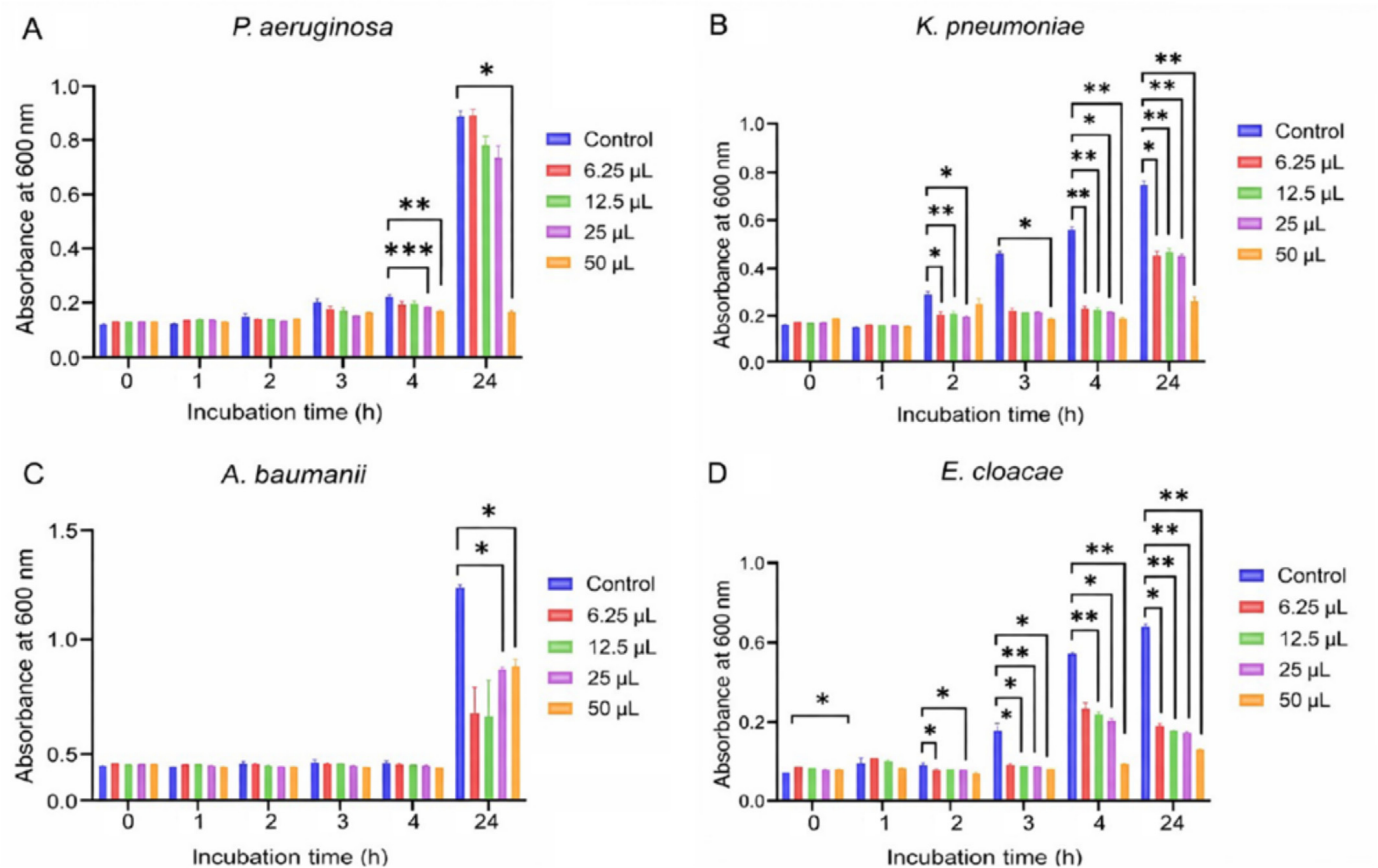
JEO inhibited the growth of clinically relevant bacterial strains. Time course bacterial growth curves were performed. JEO was tested on (A) *P. aeruginosa*, (B) mucoid *K. pneumoniae*, (C) *A. baumanii*, and (D) *E. cloacae.* Each run represents the average absorbance of triplicate readings, and the error bars represent the standard deviation. JEO: Juniper essential oil. Statistical analysis were performed using one-way ANOVA, *P < 0.05, ^**^P < 0.01, ^***^P < 0.001 *vs* control group.

JEO also demonstrated antibacterial activity against other clinically relevant ESKAPE bacterial strains including *P. aeruginosa*, *K. pneumoniae*, *A. baumanii*, and *E. cloacae*. JEO doses (50, 25, 12.5, and 6.25 µL) showed significant dose-dependent reduction in bacterial growth of *K. pneumoniae* and *E. cloacae* as early as 2 h of growth (Fig. 2B and D). Whereas, higher doses demonstrated antibacterial activity against *P. aeruginosa* and *Acinetobacer* strains (Fig. 2A and C).

To further examine the antibacterial activity of JEO it was further diluted and dissolved in 100% dimethyl sulfoxide (DMSO) then tested against ESKAPE pathogens as above. Higher doses of diluted concentrations of JEO impeded the growth of Gram-positive and Gram-negative bacterial strains (Fig. S1).

### 3.2 Antibiofilm activity of JEO

**Fig. 3:**
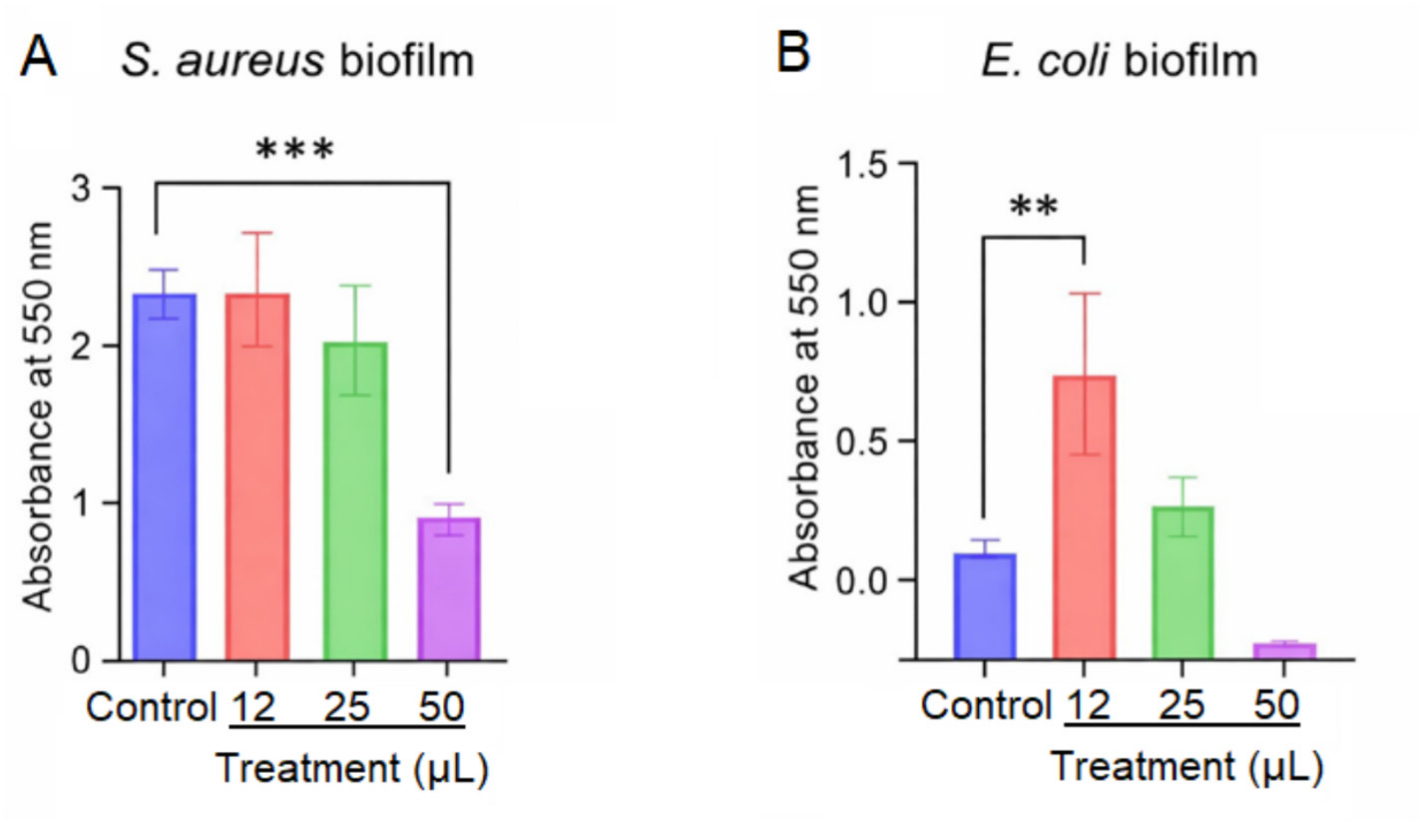
Antibiofilm activity of JEO. Biofilm formation in the presence and absence of JEO was tested on (A) *S. aureus* and (B) *E. coli,* using crystal violet staining. Each run represents the average absorbance of a quadruplicate readings, and the error bars represent the standard deviation from the mean. JEO: Juniper essential oil. Ordinary one-way ANOVA was used for statistical analysis, ^**^P < 0.01, ^***^P < 0.001 *vs* control group.

**Fig. 4:**
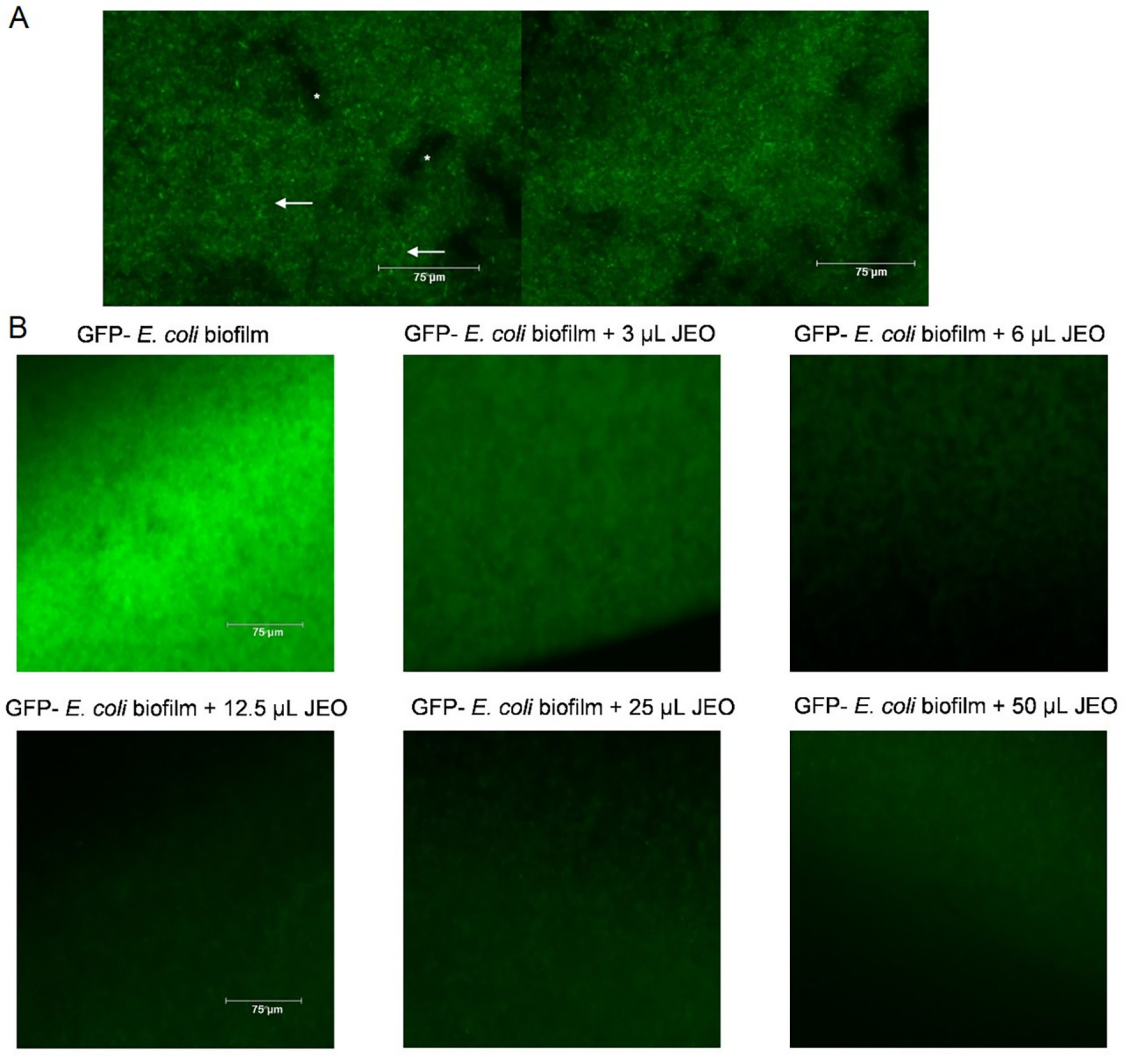
Biofilm imaging. (A) Confocal laser scanning microscopy image of GFP-labeled E. coli biofilm after 24 h of incubation, illustrating the formation of a mature 3D biofilm architecture. Distinct grooves within the biofilm matrix are marked with asterisks (*) and arrows, indicating areas of disrupted or less dense biofilm formation, likely representing channels or structural heterogeneity typical of mature biofilms. Scale bars: 75 μ m, Magnification factor: × 40. GFP: Green fluorescent protein. (B) Effect of increasing concentrations of JEO on GFP-labeled *E. coli* biofilm formation. Confocal laser scanning microscopy images show GFP-expressing *E. coli* biofilm untreated (top left) and treated with increasing volumes of JEO: 3, 6, 12.5, 25, and 50 µL. Scale bars: 75 µm, Magnification factor: ×40. JEO: Juniper essential oil. GFP: Green fluorescent protein.

Antibiofilm activity of JEO was tested against biofilm-forming pathogens *S. aureus* and *E. coli* in a dose dependent manner using 50, 25, and 12.5 µL of JEO. Biofilm formation was significantly suppressed using 50 µL of JEO in *S. aureus* and *E. coli* but not with lower doses (Fig. 3). The antibiofilm activity of JEO against GFP-labeled *E. coli* was evaluated using confocal laser scanning microscopy. Detailed biofilm imaging revealed a dense fluorescent matrix with distinct regions of reduced fluorescence intensity, indicating structural grooves or channels (Fig. 4). These features are suggestive of nutrient or oxygen diffusion pathways typical of mature biofilms and underscore the inherent complexity and spatial organization that may contribute to the biofilm’s resistance to antimicrobial agents. After 24 h of incubation, untreated *E. coli* formed a well-developed, three-dimensional biofilm structure characterized by strong and uniform green fluorescence (Fig. 4B). Upon treatment with increasing concentrations of JEO, a marked, dose-dependent decrease in fluorescence intensity was observed. As early as 3 µL JEO, a mild reduction in biofilm density became apparent, while higher concentrations (6 µL and above) resulted in progressively diminished fluorescence, indicating substantial inhibition of biofilm formation. At the highest concentration tested (50 µL), fluorescence was nearly absent, suggesting near-complete disruption of the biofilm matrix. These findings collectively demonstrate a potent, concentration-dependent antibiofilm activity of JEO against major pathogens *E. coli* and *S. aureus*.

### 3.3 Antibacterial nanofiber with JEO

**Fig. 5:**
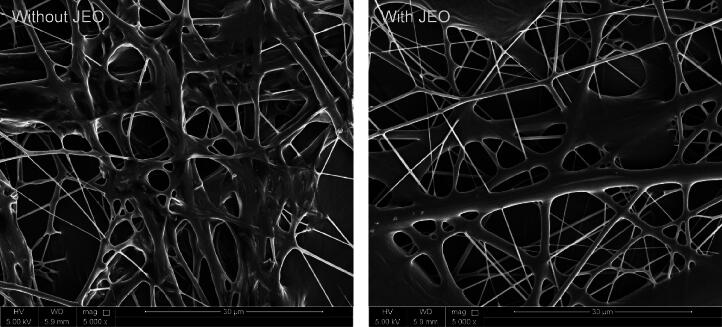
SEM images of the PCL nanofiber with or without JEO formulated with 8% JEO. JEO formulation with PCL nanofibers did not affect structure or porosity. Scale bars: 30 µm, Magnification factor: ×5000 JEO: Juniper essential oil.

**Fig. 6:**
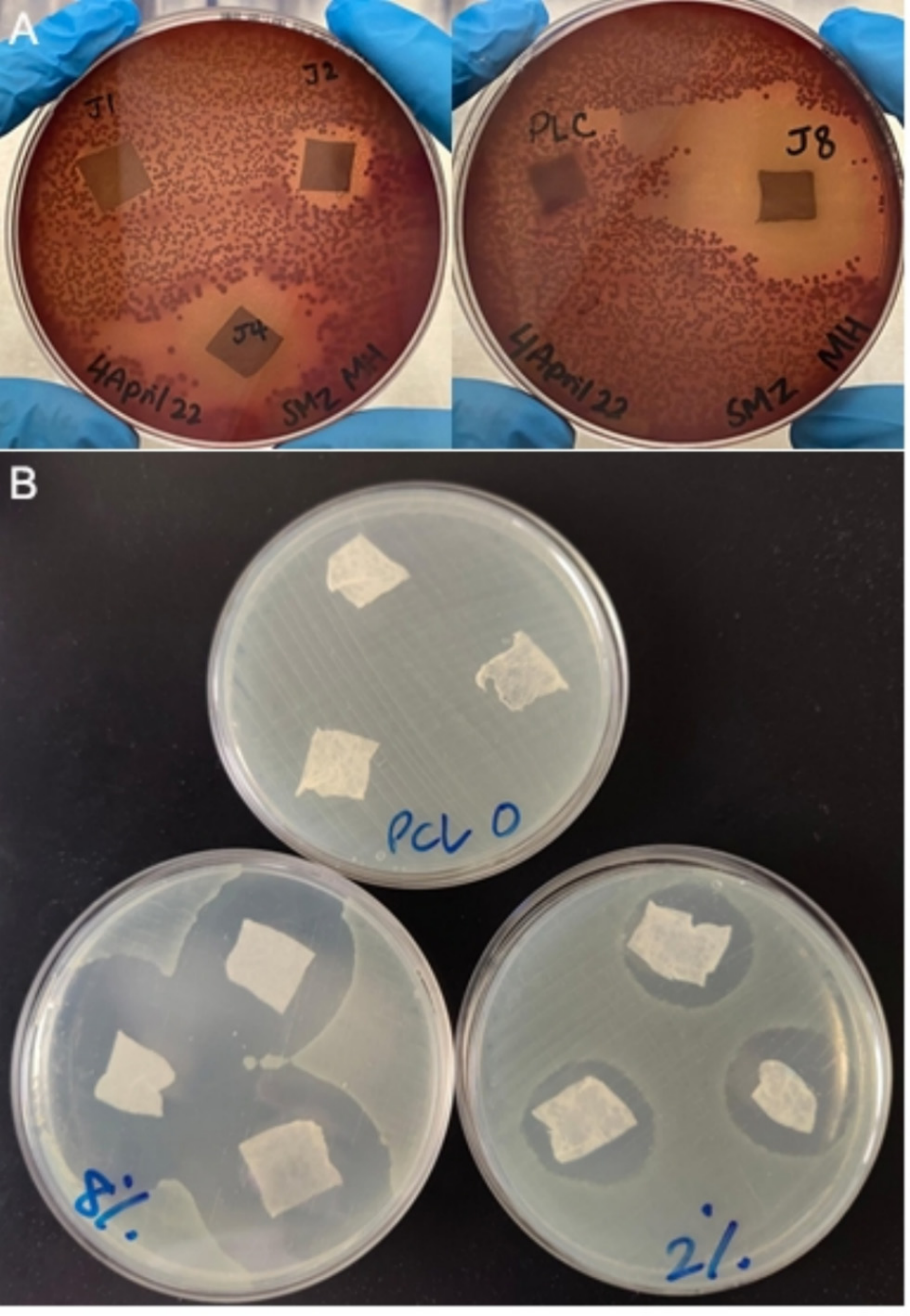
(A: Upper panel) The effect of PCL nanofibers coated with JEO on the growth of MRSA plated on sheep blood agar to assess zone of inhibition after overnight incubation at 37 °C. For each PCL nanofiber membrane, a respective amount of the JEO was tested 1% (J1), 2% (J2), 4% (J4) and 8% (J8). (B: Lower panel) PCL nanofibers coated with Juniper EO at 2% or 8% against *E. coli* plated on TSA plate. PCL nanofiber without JEO were used as controls. PCL: Polycaprolactone; JEO: Juniper essential oil; MRSA: Methicillin resistant *S. aureus*; TSA: Tryptic soy agar.

As a proof of concept, this study tested the potential use of PCL nanofibers impregnated with JEO as antibacterial bandage. The fabrication structure and porosity of PCL nanofibers were examined using scanning electron microscopy (SEM). The SEM images showed preservation of the PCL nanofiber polymers without disrupting its structure when formulated with JEO, hence suitable for antibacterial bandage application (Fig. 5). The antibacterial activity PCL nanofiber polymers impregnated with JEO doses was tested against resistant *S. aureus* strain MRSA. The highest concentration tested was 8% of JEO concentration that showed the highest activity against MRSA reflected by the largest zone of inhibition compared to lower oil percentages (Fig. 6A). Similar activity of PCL nanofibers with JEO was observed against *E. coli* (Fig. 6B).

## 4 Discussion

This study investigated the antibacterial and antibiofilm activity of PCL nanofiber bandages formulated with essential oil extracted from *J. communis* against clinically relevant bacterial pathogens commonly causing nosocomial and wound infections. JEO demonstrated a dose dependent growth inhibition in Gram-negative and Gram-positive pathogens. *J. communis* dissolved in 100% DMSO suppressed bacterial growth and replication. Moreover, antibiofilm activity of JEO was most evident at higher dose of 50 µL. PCL nanofiber membranes coated with 8% JEO coated showed the largest zone of inhibition demonstrating antibacterial activity against MRSA. Whereas, JEO did not affect the structural integrity or porosity of PCL nanofibers.

This study found a dose dependent inhibition of *S. aureus* growth at different JEO concentrations cultivated in Sardinia, Italy. The finding is in agreement with a previous study by Dumitrescu et al., where they investigated Western Romanian Carpathians JEO against *S. aureus* (Dumitrescu et al., 2022). *J. communis* is widespread across the Northern Hemisphere and has been traditionally used for its medicinal properties, particularly in treating urinary tract infections, digestive disorders, and skin ailments (Pepeljnjak et al., 2005). The JEO extracted from its berries and needles has attracted scientific interest due to its diverse biological activities, especially its antibacterial and antibiofilm properties (Raina et al., 2019). The antimicrobial effects are due to JEO components rich in monoterpenes and sesquiterpenes, which are responsible for its characteristic aroma and bioactivity. Major constituents typically include *α*-pinene, *β*-pinene, myrcene, sabinene, limonene, terpinen-4-ol, and caryophyllene (Angioni et al., 2003). These main components, particularly *α*-pinene, *β*-pinene, and sabinene, are lipophilic monoterpenes that can integrate into bacterial cell membranes. This integration disrupts bacterial membrane integrity, leading to increased permeability and eventual cell lysis. The composition can vary depending on factors such as geographical origin, part of the plant used, and extraction method (Jerković et al., 2001).

This study reported antibacterial activity of JEO against Gram-negative bacteria such as *P. aeruginosa* and *K. pneumoniae*. In contrast to our findings, Esteban and colleagues reported that *J. communis* oil from different parts of Spain lacked the ability to suppress the growth of the Gram-negative bacteria such as *K. pneumoniae* and *P. aeruginosa* (Esteban et al., 2023). These bacterial strains are part of the clinically relevant ESKAPE pathogens, responsible for significant burden of healthcare-acquired infections due to their antimicrobial resistance. ESKAPE pathogens are the leading cause of many nosocomial and community-acquired infections around the world, such as ventilator-associated pneumonia, catheter-associated urinary tract infections (CAUTI) and surgical site infections (SSIs) as well as ulcers like diabetic foot infections. *P. aeruginosa* directly causes high mortality rates in burn units where skin infections are very common and from chronic lung infections in cystic fibrosis (Lin et al., 2018). Treatment of infections caused by bacteria belonging to the ESKAPE pathogens is a clinical challenge and the selection of effective treatment strategies for these infections has become a major problem (Kawano et al., 2020, Santajit and Indrawattana, 2016). ESKAPE pathogens are associated with a higher risk of mortality and increased health care costs. The United States Centers for Disease Control and Prevention estimates that antibiotic-resistant microorganisms cause more than two million infections a year in the United States, resulting in a worldwide mortality rate of 23,000. The amount of resistance to antimicrobial agents is expected to increase 10-fold by 2050 (Ma et al., 2019).**

This study reported that JEO demonstrated a significant reduction in biofilm formation by *S. aureus* and *E. coli*. Biofilm formation in clinical settings is a significant problem as it increases resistant to antibiotics and impose challenge of eradicating nosocomial infections (Devanga Ragupathi et al., 2022). ESKAPE pathogens are notorious for their ability to form biofilms on catheters, central lines, intubations and implants (Squyres & Newman, 2024). Biofilm is a collection of bacterial cells organized in 3D structured communities forming on solid surfaces that are metabolically and physiologically different from their planktonic counterparts. Cells enclosed in the biofilm are approximately 1000-fold more resistant to the effects of antibiotics than the same cells in the planktonic state (Squyres & Newman, 2024). In addition to being protected from environmental stress, cells within the biofilm can also effectively escape the host's defense system. Biofilm is known to cause 80% of refractory infections such as chronic lung infections, wound infections and ear infections (Squyres & Newman, 2024). Bacteria easily forms biofilms on medical devices such as catheters and implants, thereby increases hospitalization and treatment costs (Muhammad et al., 2020, Sur et al., 2004). The antimicrobial and antibiofilm activity of JEO may be beneficial in the management of antibiotic-resistant pathogens (Kerekes et al., 2013).

The continued rise in AMR infections caused by ESKAPE pathogens especially in healthcare settings prompts actions to mitigate the threat of treatment failure and spread of nosocomial infections. Catheters and open wounds represent high-risk environments for colonization by AMR pathogens, contributing to prolonged wound healing, elevated morbidity and mortality rates, and prolonged hospitalizations, thereby imposing substantial economic burden (Bertagnolio et al., 2024). The development of novel nanofiber bandages aid in both preventing infection spread and expediting wound healing process, consequently mitigating the dissemination of AMR pathogens. Such advancements not only have the potential to reduce mortality, but also alleviate healthcare expenditures, thereby easing the strain on healthcare infrastructures while promoting enhanced patient outcomes. New antimicrobial agents should emerge to reduce the prevalence of antimicrobial resistant bacterial infections. There is a low frequency of infections in wild plants, indicating that utilized natural defense mechanisms can be very effective, which promote the use of plant-derived medicines such as essential oils (Abdallah et al., 2023).

For medical purposes, the plant-derived essential oils are usually used as topical applications, not oral or parenteral routes. It was shown that natural antimicrobials are capable of decreasing the minimum inhibitory concentration of antibiotics in several groups of bacteria that contain genetic elements responsible for drug resistance (Palaniappan & Holley, 2010), thus JEO can act as an antibiotic adjuvant. JEO is generally considered safe, however, certain precautions are necessary when considered for medicinal use. Dermal irritation may occur, thus diluted formulation is suggested for topical applications (Tisserand & Young, 2014). Additionally allergic reactions may occur in individuals with allergies to coniferous plants (Tisserand & Young, 2014). Internal use of JEO is not preferred, due to its potential nephrotoxicity at high doses (Tisserand & Young, 2014).

Antibiotics are usually applied directly on infected skin areas such as wounds, ulcers or SSIs. Bandages are usually used to cover wounds and placed over the layer of topical antibiotic ointment when indicated. Recent advances in novel medical bandages development showed a proof-of-concept for biological bandage with antibacterial and/or anti-inflammatory material to accelerate wound healing process and prevent infection (Abdel-Sayed et al., 2019). Novel biological bandages are formulated as films, hydrogels, and nanocomposites gauze-like material (Vivcharenko and Przekora, 2021, Wojcik et al., 2021). PCL-based materials have been widely used in wound healing applications (Raina et al., 2022). This study demonstrated the use of electro-spun PCL-based nanofibers material as wound bandage formulated with JEO possessing antibacterial and anti-biofilm activity (Augustine et al., 2015, Hasan et al., 2018). While direct studies on JEO specifically for wound healing are scarce, the anti-inflammatory properties of its constituents may aid in the healing process. Terpinen-4-ol, a component found in JEO, has been noted for its anti-inflammatory effects (Orchard & van Vuuren, 2017). Reducing inflammation can promote a more favorable environment for wound healing (Orchard & van Vuuren, 2017). Utilizing plant-derived essential oils such as JEO as medications has limitations, mainly due to volatile hydrophobic nature, i.e. is not miscible in water, therefore technical applications in aqueous media would require formulations with other additives to solubilizes the oil, without affecting antibacterial activity. Further research is needed to prepare stable JEO formulations suitable for medical application.

This study is a proof-of-concept that has several limitations. First, while the antibacterial and antibiofilm effects of JEO were observed, no compositional or quality analysis of the essential oil was available. Since extraction methods and chemical compositions are critical for standardization and reproducibility, the absence of GC–MS profiling, yield data, or physicochemical characteristics limits both repeatability and the ability to correlate specific constituents with biological activity. Additionally, the standardization process of the JEO extraction is not described, further impacts reproducibility. Second, although JEO was incorporated into PCL nanofibers via electrospinning, the preparation method and key physicochemical parameters including fiber diameter, thickness, mechanical strength, and essential oil loading content, were not evaluated. These data are necessary to assess formulation quality and performance, and warrant future thorough investigation. Third, statistical validation of the *in vitro* antibacterial activity of the JEO-loaded nanofibers was not performed due to the preliminary nature of the experiment and limited sample replicates. Lastly, the observed differences in antibiofilm sensitivity among different bacterial strains were not explored in depth. These variations may stem from strain-specific factors such as biofilm matrix architecture, efflux mechanisms, or quorum sensing systems, which influence essential oil susceptibility. Future studies should address these limitations by including full essential oil characterization, comprehensive nanofiber formulation analysis, appropriate statistical methods, and deeper exploration of strain-dependent responses to essential oil treatments.

## 5 Conclusion

JEO exhibits antibacterial and antibiofilm properties against ESKAPE pathogens. Utilizing JEO within PCL nanofiber bandages holds promise for addressing surgical site infections and skin ulcers in wound care. Further studies are required to optimize JEO pharmaceutical formulations that are suitable for clinical care.

## CRediT authorship contribution statement

**Mohannad N. AbuHaweeleh:** Methodology, Formal analysis, Data curation, Investigation, Visualization, Writing – original draft, Writing – review & editing. **Ahmad Hamdan:** Methodology, Formal analysis, Data curation, Investigation, Visualization, Writing – original draft, Writing – review & editing. **Menatalla Metwally Said:** Methodology, Formal analysis, Data curation, Investigation, Visualization, Writing – original draft. **Maryam Hassiba:** Methodology, Formal analysis, Data curation, Investigation, Visualization, Writing – original draft. **Robin Augustine:** Conceptualization, Writing – original draft, Writing – review & editing. **Anwarul Hassan:** Writing – original draft, Writing – review & editing. **Nahla O. Eltai:** Writing – original draft, Writing – review & editing. **Susu M. Zughaier:** Conceptualization, Methodology, Formal analysis, Data curation, Investigation, Visualization, Writing – original draft, Writing – review & editing.

## Declaration of competing interest

The authors declare that they have no known competing financial interests or personal relationships that could have appeared to influence the work reported in this paper.

## Appendix A Supplementary data

Supplementary Data 1 Figure S1. Antibacterial activity of diluted JEO on bacterial growth curves. S. aureus (A), MRSA (B), KPS (C), and KPR (D) were treated with JEO dissolved in 100% DMSO and monitored for 4 hours. The absorbance represents the average of triplicate wells reading, and the error bars represent standard deviation from the mean. Ordinary one-way ANOVA was used for statistical analysis, * p <0.05, ** p <0.01, *** p <0.001.
