# Supplementary material for: Antibacterial and antibiofilm activity of novel nanofibers bandage formulated with Juniperus communis essential oil targeting antibiotic resistant bacterial strains
Source: Chin Herb Med. 2026 May 8;18(3):758–66. doi: 10.1016/j.chmed.2026.05.005 (PMC13390023; doi:10.1016/j.chmed.2026.05.005)
Supplement: Supplementary Data 1 — Figure S1. Antibacterial activity of diluted JEO on bacterial growth curves. S. aureus (A), MRSA (B), KPS (C), and KPR (D) were treated with JEO dissolved in 100% DMSO and monitored for 4 hours. The absorbance represents the average of triplicate wells reading, and the error bars represent standard deviation from the mean. Ordinary one-way ANOVA was used for statistical analysis, * p <0.05, ** p <0.01, *** p <0.001. [file mmc1.doc]

**Supplementary materials**

**A****ntibacterial and antibiofilm activity of novel nanofibers bandage formulated with *Juniperus communis* essential oil targeting antibiotic resistant bacterial strains**

Mohannad N. AbuHaweeleh ^a,1^, Ahmad Hamdan ^a,1^, Menatalla Metwally Said ^a^, Maryam Hassiba ^a^, Robin Augustine ^b^, Anwarul Hassan ^c^, Nahla O. Eltai ^d^, Susu M. Zughaier ^a,*^

^a^ Department of Basic Medical Sciences, College of Medicine, QU Health, Qatar University, Doha 2713, Qatar

^b^ Department of Radiology, Stanford Medicine, Stanford University, CA 94304, USA

^c^ Department of Mechanical and Industrial Engineering, College of Engineering, Qatar University, Doha 2713, Qatar

^d^ Biomedical Research Center, QU health, Qatar University, Doha 2713, Qatar

*Corresponding author.

*E-mail address*: [szughaier@qu.edu.qa](mailto:szughaier@qu.edu.qa) (S. M. Zughaier).

^1^ These authors contributed equally to this work.

**
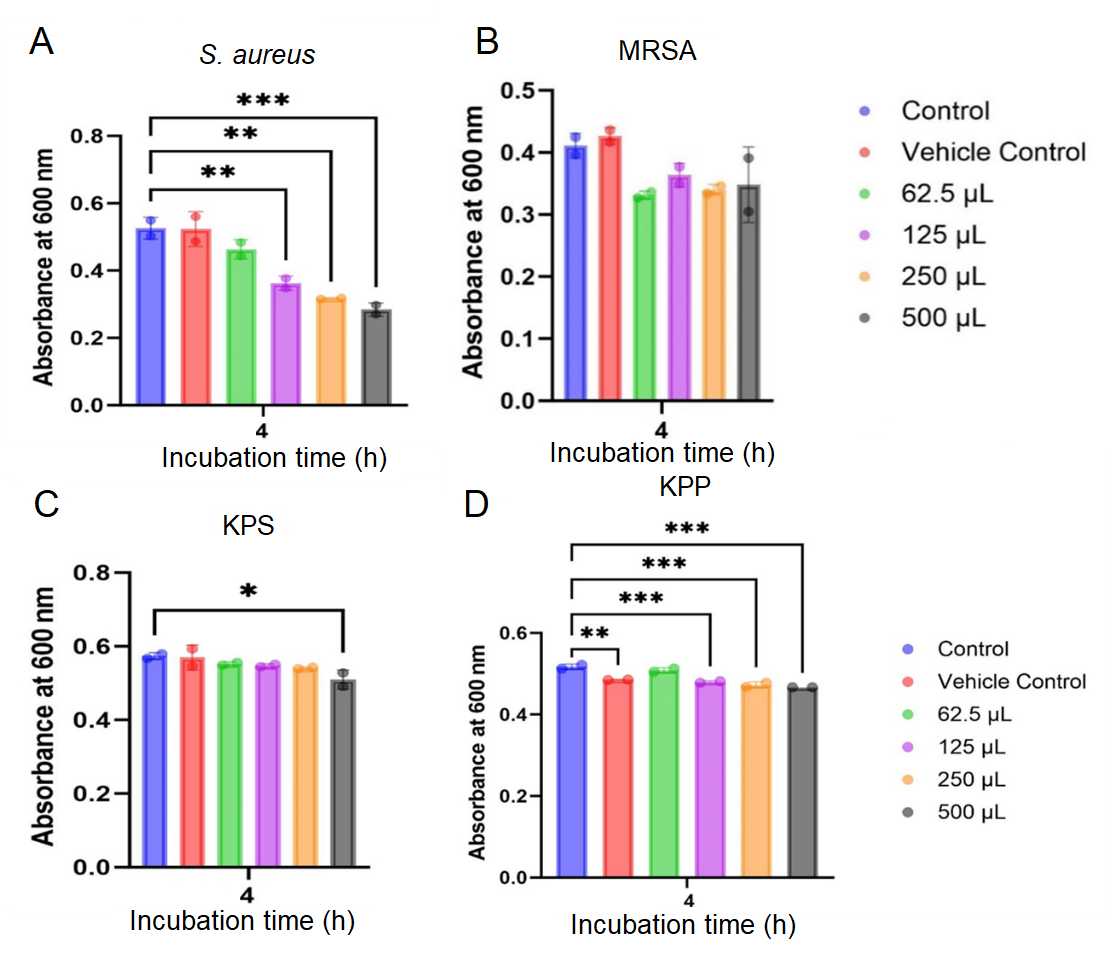
**

**Fig. S1.** Antibacterial activity of diluted juniper oil on bacterial growth curves. *S. aureus* (A), MRSA (B) and KPS (C) and KPR (D) were treated with Juniper EO dissolved in 100% dimethyl sulfoxide (DMSO) and monitored for 4 h. The absorbance represents the average of triplicate wells reading, and the error bars represent standard deviation from the mean. Ordinary one-way ANOVA was used for statistical analysis, ^*^*P* <0.05, ^**^*P* <0.01, ^***^*P* < 0.001 *vs* control group.
